# C1-inactivator is upregulated in glioblastoma

**DOI:** 10.1371/journal.pone.0183086

**Published:** 2017-09-07

**Authors:** Karolina Förnvik, Aida Maddahi, Oscar Persson, Kurt Osther, Leif G. Salford, Henrietta Nittby Redebrandt

**Affiliations:** 1 The Rausing Laboratory, Division of Neurosurgery, Department of Clinical Sciences Lund, Lund University, Lund, Sweden; 2 Department of Neurosurgery, Karolinska University Hospital, Stockholm, Sweden; 3 Osther Consulting, LLC, Scottsdale, Arizona, United States of America; Thomas Jefferson University, UNITED STATES

## Abstract

**Background:**

Glioblastoma is the most common and aggressive type of primary brain tumor in adults. A key problem is the capacity of glioma cells to inactivate the body’s immune response. The complement system acts as a functional bridge between the innate and adaptive immune response. Still, the role of the complement system has almost been forgotten in glioma research. In our present study, we hypothesize that C1 inactivator (C1-IA) is upregulated in astrocytoma grade IV, and that its inhibition of the complement system has beneficial effects upon survival.

**Methods and results:**

We have explored this hypothesis both on gene and protein levels and found an upregulation of C1-IA in human glioblastoma cells using data from a publicly available database and our own mRNA material from glioblastoma patients. Furthermore, we demonstrated the presence of C1-IA by using immunohistochemistry on glioma cells from both humans and rats in vitro. Finally, we could demonstrate a significantly increased survival in vivo in animals inoculated intracerebrally with glioma cells pre-coated with C1-IA antibodies as compared to control animals.

**Conclusions:**

Our findings indicate that overexpression of C1-IA is present in glioblastomas. This could be demonstrated both at the gene level from patients with glioblastoma, on mRNA level and with immunohistochemistry. Treatment with antibodies against C1-IA had beneficial effects on survival when tested in vivo.

## Introduction

Glioblastoma is the most common and aggressive type of primary brain tumor in adults. There are currently no curative ways to treat these malignant tumors, even though patients undergo multimodality treatment with surgery, chemotherapy and radiotherapy [1]. One of the key problems when treating patients with malignant gliomas is, that despite being able to remove the major bulk of the tumor through neurosurgery, malignant tumor cells have already spread throughout the brain when the patients undergo the operation, and even extensive resections will not cure the patient. Another key problem is the capacity of glioblastoma cells to inactivate the body’s immune response against them. Much attention has been drawn to the T-cell dependent immune response, where inactivation of cytotoxic T-cells and increase in regulatory T-cells has been seen. Immune therapies have shown some promise regarding increased survival in patients with glioblastoma, but no major breakthrough has been described and still, there is no cure against glioblastomas [2].

The complement system is another part of the immune response, which has been less studied in the glioma research. However, its function may be essential, and therefore, we have investigated the role of complement inactivation in glioma. Under normal conditions, the complement system is a functional bridge between the innate and the adaptive immune response [3]. Three components in this system have been identified; the classical pathway, the alternative pathway and the lectin induced pathway. These pathways all converge at the level of C3 convertase. The classical pathway is initiated by activation of the C1 complex, consisting of one molecule of C1q, two molecules of C1r and two molecules of C1s.

The main function of complement 1 inactivator (C1-IA) is to inhibit activation of the complement system through the classical pathway, by irreversibly binding to and inactivating of C1r and C1s proteases in the C1 complex of the classical pathway. It is the only known physiological inhibitor of C1s and C1r, the activated homologous serine proteases of the first component of the complement, thus playing an essential role as a regulator of complement classical pathway in blood and tissue [4]. In the 1970ies, it was demonstrated that human primary and secondary malignant brain tumors were coated with a protein, immunologically indistinguishable from complement component C1-IA. The investigations were done by immunofluorescence cytophotometry of individual cells cultured from human brains [5].

In our present study, we hypothesize that the classical pathway of the complement system is inactivated in glioblastoma through the upregulation of C1-IA, and furthermore, we hypothesize that activation of the complement system is beneficial. The findings by Osther et al. [5] support our hypothesis, and so do more recent findings. H2 glioma cells have turned out to be very resistant to complement-mediated cytolysis in vitro. Experiments have shown that H2 cells strongly express the membrane attack complex inhibitor protectin (CD59) and that H2 cells actively produce the soluble complement inhibitors factor H and factor H-like protein 1 [6].

We have investigated the expression of C1-IA in human glioblastoma cells using data from a publicly available database. We also investigated our own material [7] with mRNA data from patients with glioblastoma, to see whether our findings could confirm these findings. Furthermore, we investigated glioblastoma cells from both humans and rats, to see if C1-IA proteins could be identified in vitro with immunohistochemistry. Finally, we tested our hypothesis in vivo, by using a Fischer 344 rat model, where GFP-expressing NS1 glioblastoma cells were inoculated intracranial after coating the cells with antibodies against C1-IA.

## Materials and methods

### Gene expression analysis

Two different sets of data were analyzed for mRNA gene expression levels of complement associated genes. The first set of data was derived from previously published material from our own institution [7]. This data set used 27 k in-house printed cDNA microarrays to investigate global gene expression changes in non-tumor samples from four patients with intractable epilepsy and compared to high-grade glioma (astrocytoma WHO grade IV) from 26 patients. Tissue samples were processed and analyzed as previously described [7]. All handling of patient material and data was approved and performed according to the guidelines of the Regional Ethics Review Board in Lund.

The second set of data was retrieved from a publicly available database (GSE4290 Affymetrix Gene Chip Analysis; Sun L, Hui AM, Su Q, Vortmeyer A et al. Neuronal and glioma-derived stem cell factor induces angiogenesis within the brain. Cancer Cell 2006 Apr;9(4):287–300. PMID: 16616334; https://www.ncbi.nlm.nih.gov/geo/query/acc.cgi?acc=GSE4290). This set of data was performed using the Affymetrix Human Genome U133 Plus 2.0 Array platform to investigate global gene expression levels in 23 non-tumor brain samples and 77 samples from patients with glioblastoma.

### Human glioma cell lines

Three primary glioma cell cultures (AMN, DZ and GA) established as previously described [7] were used. Briefly, tumor tissue was retrieved intra-operatively after approval of the Regional Ethics Review Board in Lund. Tumors were suspended as single-cells and cultured in Iscove’s Modified Dulbecco’s Medium (IMDM) supplemented with 20% fetal calf serum, 1% penicillin streptomycin, and 1% sodium pyruvate in minimal essential medium. The cells were regularly karyotyped until they consistently exhibited a dominating tumor-specific karyotype.

### Rat glioma cells

The rat glioma cell lines RG2 and NS1 [8] were used. Both were derived from ethylnitrosurea-induced glioma in Fischer 344 rats and used in fully immunocompetent rats. RG2 was produced in Fischer 344 rats through ENU treatment of pregnant females [9]. RG2 is a particularly aggressive model with short survival from tumor cell inoculation to presentation of symptoms due to aggressive tumor growth (19.4 ± 3.8 days) [10]. RG2 is non-immunogenic in syngeneic Fischer rats [11]. Since the RG2 is a relatively old tumor model, we used our own newly developed NS1 model for in vivo studies [8].

NS1 is a new GFP positive tumor cell line which was created by ENU treatment of pregnant homozygous GFP-positive Fischer 344 rats [8], where the offspring developed GFP-positive CNS-tumors, resulting in the NS1 cell line. Rats inoculated with NS1 cells develop cell-rich tumors with an invasive growth pattern, and since the tumors are GFP-positive, the infiltrative pattern can be studied. The tumors are positive for GFAP, GFP and the tumor cells have been shown to have a strong RNA expression for wt IDH1, wt p53, IDO1 and EGFR.

### Cell culture

The rat glioma cells (NS1) were cultured using RPMI-1640 (Sigma-Aldrich) medium with addition of 1% ml Na-pyruvate, 1% ml HEPES (4-(2-hydroxyethyl)-1-piperazineethanesulfonic acid), 0.1% ml gentamycin, as well as 10% inactivated fetal calf serum (heated to 56°C for 30 minutes). After culturing in T25 flasks, the cells were prepared for inoculation by removal of the medium and washed gently with PBS. Trypsin el TrypleTM Express (Invitrogen) was added and cells were incubated in 37°C for 1–2 minutes to detach the adherent cells from the flask. More medium was added and viable cells were counted. The cells were centrifuged at 1200 rpm for 5 minutes at 4°C, then the supernatant was carefully removed to avoid any potentially immunogenic calf serum. Afterwards the cell pellet was resuspended in serum-free medium (R0) to achieve the concentration used for inoculation, 1000 cells/μl.

### Immunohistochemistry

Human glioma cells from three of the patients (AMN, DZ and GA) were used in this present study to evaluate protein expression of C1-IA in vitro. From rats, RG2 and GFP positive NS1 glioblastoma cells were used. The cells were cultured for 1–2 days in two-chamber culture slides (Thermo Fisher Scientific) at 37°C in a humidified 5% CO2 incubator. The medium was carefully removed and the cells were fixed in 4% paraformaldehyde for 20–30 minutes at room temperature. The cells were then washed three times with phosphate buffer solution (PBS) and blocked for 1 hour using a blocking solution containing PBS, 1% bovine serum albumin (BSA) and 5% normal goat serum, and then incubated overnight at 4°C with rabbit anti-human C1 inactivator (LS-C192710/66843, LifeSpan BioSciences, Sweden) or rabbit anti-rat C1 inactivator (Covance; USA). The primary polyclonal antibodies were diluted 1:400 in PBS containing 1% BSA and 2% normal goat serum. The cells were subsequently incubated for 1 hour at room temperature with secondary antibodies; consisting of FITC-conjugated goat anti-rabbit (F9887, Sigma), diluted 1:200 or Alexa Fluor 594-conjugated goat anti-rabbit (ab150084, Abcam) diluted 1:400 in PBS containing 1% BSA. After washing with PBS, the cells were mounted with anti-fading vectashield mounting medium with 4,6-diamidino-2-phenylindole (DAPI) (Vector Laboratories Inc., Burlingame, USA) and were photographed with a fluorescent microscope fitted with the appropriate wavelength filters. The same procedure was used for the negative controls except that either the primary antibody or the secondary antibody was omitted to verify that there was no auto-fluorescence or unspecific labeling or binding.

### In vivo experiments

Fischer 344 rats were used (Fischer Scientific, Germany). These rats were housed in pairs in rat cages with access to water and fed ad libitum with rat chow. The animals were monitored daily, and those displaying signs of paresis, epilepsy, or declined general condition were euthanized. If an animal should survive for 100 days without displaying symptoms, it would be euthanized in accordance with the ethical permission. This study was approved by the animal ethics committee in Lund with permit ID M102-16. All efforts were made to minimize animal suffering.

Each rat received an intracranial inoculation of 5000 cells from the GFP tumor line, suspended in 5 μl of nutrient solution. Intracerebral tumor cell inoculation was done under isoflurane inhalation anesthesia using a stereotactic frame and a 10 μl Hamilton syringe. The cells were injected at a depth of 5 mm, 2 mm laterally from the sagittal suture, and at the coronary suture, on the right side of the cranium. The cranial burr hole was sealed with bone wax, and the incision was closed with absorbable suture.

The experiments consisting of pre-treatment with C1-IA coated tumor cells by incubating the cells with rabbit anti-human C1-IA antibodies to coat the cells (the cells are known to cross react with rat C1IA) or by using rabbit anti-rat C1-IA antibodies, for two hours at 37°C directly prior to intracranial inoculation. This was done at a ratio of 1/5 with a final cell concentration of 1000 cells/μl. Control cells were not pre-treated with antibodies.

Animals were examined and observed daily and were euthanized immediately if they started to show any neurological symptoms, upon which the brains were removed. The brains were fixed in isopenthane and cryosectioned, after which they were stained with hematoxylin-eosin.

### Statistical analysis

All gene expression values were log2-transformed and statistical analysis was performed using IBM® SPSS® Statistics ver 20.0.0. Calculation of group mean differences and confidence intervals was performed using t-test without assuming equal variance. Gene expression alteration were considered to be statistically significant for fold change > 2 and p-value < 0.05.

In vivo experiments were evaluated using 2-tailed student’s t-test assuming equal variance.

## Results

### Increased activity of C1-IA in glioblastoma

**Fig 1:**
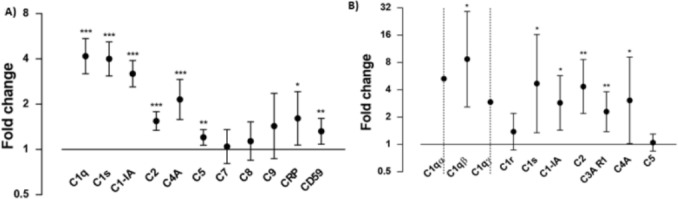
Gene expression analysis showing upregulation of complement associated genes in two independent data sets. Figs show fold change of group mean expression values with 95% confidence interval for astrocytoma grade IV vs. non-tumor brain in a data set from a publicly available database (Fig 1A) and our own institution (Fig 1B). Confidence intervals for C1α and C1qɤ lie outside the scale. Significance levels are indicated as *p<0.05, **p<0.01, and ***p<0.001.

To evaluate whether complement inactivation was present in glioblastoma, we quantified the gene expression of complement factors in tissue from human tumors using data from a publicly available database (GSE4290 Affymetrix Gene Chip Analysis). This revealed a significantly higher expression with a fold change of 3.2 of C1-IA (p < 0.001) in glioblastoma tissue as compared to non-tumor tissue (S1 Fig and Fig 1A). Since C1-IA inhibits the C1 complex from forming multifaceted inflammatory effects in various animal models and human diseases [4], the sub-components of the C1 complex were also upregulated. However, the final downstream regulators in the cascade were not upregulated. That is, C1q (fold change 4.2; p < 0.001), C1s (; fold change 4.0; p < 0.001) and C4a (fold change 2.1; p < 0.001) were significantly up-regulated in material from patients with glioblastoma as compared to non-tumor controls.

Next, we were able to confirm these findings by the analysis of mRNA data from our own patient material, where a significant upregulation of C1-IA (fold change 2.9; p = 0.01) could be found in patients diagnosed with glioblastoma, when compared to expression in tissue from patients undergoing epilepsy surgery (Fig 1B). The same pattern of upregulation of the components of the C1 complex was observed as in the Affymetrix data and C1s (fold change 4.7; p = 0.027) and C1qβ(fold change 8.7; p = 0.011) were upregulated in glioblastoma patients as compared to controls.

**Fig 2:**
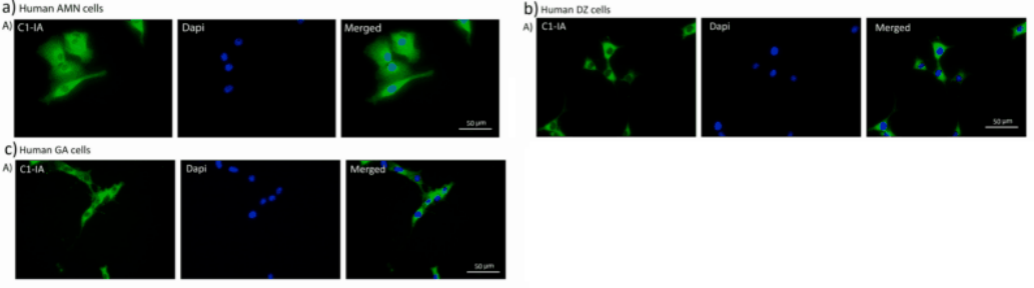
Demonstration of C1-IA at the protein expression level in human glioblastoma cells. The antibody binding seems to be localized to the cell membrane and cytoplasm.

In order to confirm the protein expression further, cells from three of the patients with glioblastoma were analyzed with immunohistochemistry. The cells were stained with human anti-C1-IA and subsequently with secondary FITC labeled sandwich antibody. Also here, C1-IA could be demonstrated (Fig 2A–2C).

### Effect of complement activation on survival in an intracranial tumor model

In order to test the role of complement re-activation in glioblastoma, we needed a fully immunocompetent animal model. Firstly, we wanted to test the expression of C1-IA in both an old model, the ENU-induced RG2; and our own ENU-induced GFP-positive NS1 model.

**Fig 3:**
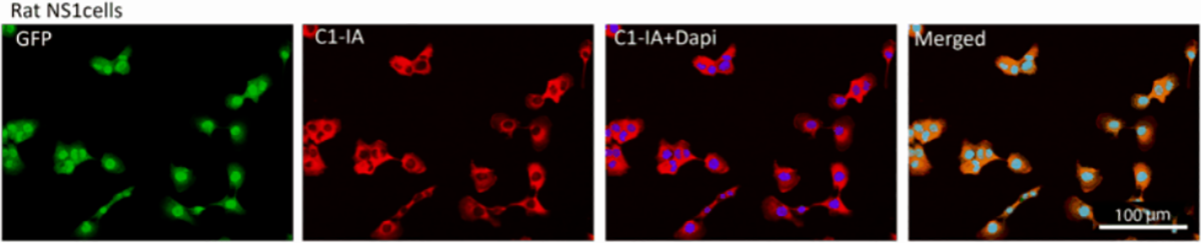
Also in our own rat glioblastoma cell line with homozygously GFP-positive cells, C1-IA could be demonstrated both A) with the human anti-C1-IA and B) with the rat anti-C1-IA.

**Fig 4:**
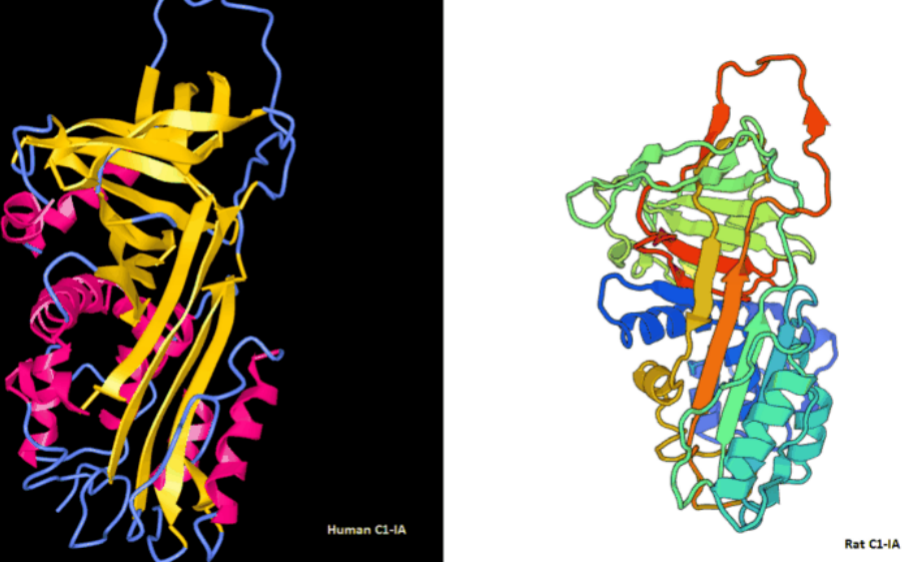
Protein structure for (left) human C1-IA and (right) rat C1-IA (model from Swissmodel.expasy.org). The human C1-IA consists of 500 amino acids, whereas the rat C1-IA consists of 504 amino acids with a sequence identity of 78.57%.

The complement inactivation in rat glioblastoma cells was verified with immunohistochemistry in the same fashion as done with the human glioblastoma cells, where C1-IA could be demonstrated in rat glioma cell lines, both the old RG2 line and our new GFP positive glioma cell line (NS1) with anti-C1-IA antibodies and secondary FITC labeled sandwich antibody, both with the rabbit-anti human C1-IA and the rabbit-anti-rat C1-IA (Fig 3A and 3B). In our experiments we could see that antibodies both targeted against human C1-IA and rat C1-IA could bind to rat glioma cells. It is known that the C1-IA protein is quite conserved between the two species, which further strengthens the impression that this plays a very important role in the immune response. This could also be confirmed when analyzing the protein structure for C1-IA in humans and rats (Fig 4 and data in S1 Text).

Since it is now known that the old cell lines, like the RG2 cell line, might have be altered through many years of culturing, and therefore might not represent a perfect model for glioblastoma, we wanted to test the treatment with complement activation in our own new NS1 model, where all the tumor cells homozygously express GFP and form invasive tumors. These cells can be injected into fully immunocompetent, syngeneic Fischer 344 rats, with a mean survival of 18 days after inoculation of 5000 cells into the right caudate nucleus (data not shown). The major advantage is that the rats express a fully competent immune response, as compared to mice used in xenograft models. Since the immune response was the topic to be studied, we chose to test the effect of anti-C1-IA therapy in vivo using our NS1 tumor model (S2 Fig).

**Fig 5:**
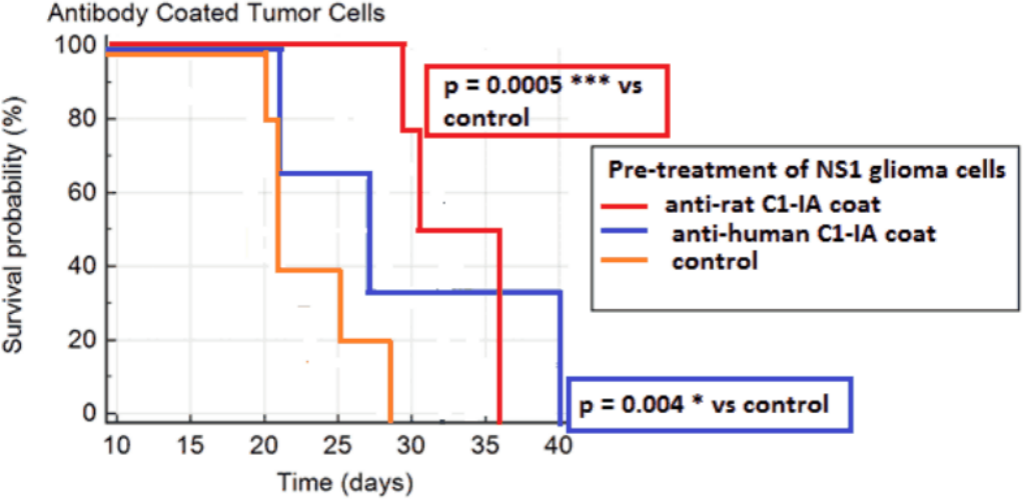
Animals inoculated with glioblastoma cells coated with anti-C1-IA antibodies had a significantly longer mean survival as compared to the control animals. No side effects of antibody coating was seen.

We evaluated whether re-activation of the complement system could affect survival of rats with NS1 glioblastomas. The GFP positive NS1 cells coated with rabbit anti-human C1-IA or no antibodies (controls) were stereotactically injected into the caudate nucleus of fully immunocompetent Fischer 344 rats. Mean survival ± SD in the control animals was 23.0 ± 3.4 days (n = 5), as compared to 29.7 ± 4.0 days (n = 3) in the animals with cells coated with C1-IA antibodies. Survival was significantly increased in the rats with anti-C1-IA coated glioma cells as compared to control animals (2 –sided t-test p-value = 0.045) (Fig 5).

Finally, the rabbit anti-rat C1-IA antibodies were used to pre-coat the NS1 cells. Under these conditions, mean survival in animals treated with pre-coated cells was 32.8 ± 3.8 days (n = 4). Also here, survival was significantly increased as compared to control animals (2-sided t-test p-value = 0.0045). Histochemistry demonstrated the growth of an infiltrative tumor in the brains.  

## Discussion

We here stipulated that the C1-IA, a potent inhibitor of the classical pathway of the complement system, was upregulated in high grade glioblastomas. In the present study we could demonstrate a consistent upregulation of C1-IA in glioblastoma as compared to the non-tumor situation. Affymetrix data from a publicly available database, our own mRNA data from glioblastoma patients, and immunohistochemistry from both human and rat glioblastoma cells confirmed our hypothesis. Furthermore, C1-IA expression could be demonstrated with immunohistochemistry. We could demonstrate a survival benefit in animals with glioblastoma cells pre-treated with anti-CI-IA antibodies. Taken together, this strengthens the hypothesis that C1-IA is upregulated in glioblastoma and that inhibiting this protease inhibitor with antibodies seems to have beneficial effects.

The gene analysis revealed increased expression of C1q and C1s in glioblastomas. This increase is believed to be a result of the decreased formation of the C1qr2s2 complex due to the increased level of C1-IA. Furthermore, CRP was significantly upregulated on the gene level in patients with glioblastoma. Previous research has shown that elevated levels of CRP preoperatively in patients with high grade astrocytoma is correlated to decreased survival [12]. The role of CRP in high grade astrocytomas would be interesting to explore further in future research.

In the past, some therapeutic strategies have focused on inhibiting the complement-mediated response against tumors, since a tumor-promoting role was suspected, where complement activation was thought to support chronic inflammation, promote an immunosuppressive microenvironment, induce angiogenesis, and activate cancer-related signaling pathways [13]. This led to additional investigations of the complement inhibitors, which are grouped into two categories, soluble regulators and membrane-bound regulators. Bouwens et al. [14] showed a diffuse staining of C3 in GBM tumor tissue. However, C5b-C9 complex was detected only in individual cells, meaning that only some local activation of this complex had taken place. One problem with these previous studies was that the role of complement inhibitory proteins was not fully addressed. Recently, it has been suggested that activating the complement system with monoclonal antibodies might serve as an important function in targeting malignancies [15]. Furthermore, studies have shown that high levels of pre-operative C1 inactivator is correlated to early cancer recurrence [16].

## Conclusions

Taken together, we have here put forward the hypothesis that in fact, the classical pathway of the complement system is inactivated in the glioblastoma setting. We have demonstrated increased levels of C1-IA on both the gene and protein expression levels from patients with glioblastoma. We could see the same pattern with immunohistochemistry in cells from humans and rats; and finally we showed that treating rats with pre-coated tumor cells treated with anti-C1-IA prolonged survival in our fully immunocompetent rat glioblastoma tumor model in vivo.

## Supporting information

S1 Fig Schematic drawing of the complement reaction, showing (left) the effect of C1-IA and (right) re-establishing the reaction by anti-C1-IA treatment. (TIF)

S2 Fig The NS1 tumor model, showing the NS1 tumor in fluorescent microscope after inoculation into a syngeneic Fischer 344 rat. (TIF)

S1 Text Protein structure for C1-IA in humans and rats. (DOCX)
