# Supplementary material for: C1-inactivator is upregulated in glioblastoma
Source: PLoS One. 2017 Sep 7;12(9):e0183086. doi: 10.1371/journal.pone.0183086 (PMC5589086; doi:10.1371/journal.pone.0183086)
Supplement: S1 Text — (DOCX) [file pone.0183086.s003.docx]

**S1 Text.** It is known that the C1-IA protein is quite conserved between humans and rats, which further strengthens the impression that this plays a very important role in the immune response. This could also be confirmed when analyzing the protein structure for C1-IA in humans and rats.

Sequence rat C1-IA:

MASKLTPLTL LLLLLAGDRA FSDSEVTSHS SQDPLVVQEG SRDSVPERDG

60 70 80 90 100

SRSPIEHTGQ SSTWPTTSGS TKISNDTMDQ VANESFIQHV QPAAQLPEDS

110 120 130 140 150

PSQSPVNSSS PPSTASAPPT QAPTEPLCPE PLAWCSDSDR DSSEATLSEA

160 170 180 190 200

LTDFSVKLYH AFSATKKAET NMAFSPFSIA SLLTQVLLGA GDSTKSNLED

210 220 230 240 250

ILSYPKDFAC VHQTLKAFSS KGVTSVSQIF HSPDLAIRDT YVNASLSLYG

260 270 280 290 300

SSPRVLGPDG DANLKLINTW VAENTNHKIN ELLDSLPSDT RLVLLNAVYL

310 320 330 340 350

SAKWKKTFEQ KKMMASFLYK NSMIKVPMLS SKKYPLALFN DQTLKAKVGQ

360 370 380 390 400

LQLSHNLSFV IMVPQSPTHQ LEDMEKALNP TVFKAILKKL ELSKFQPTYV

410 420 430 440 450

MMPRIKVKSS QDMLSIMEKL EFFDFTYDLN LCGLTEDPDL QVSSMKHETV

460 470 480 490 500

LELTETGVEA AAASTISVAR NLLIFEVQQP FLFLLWDQRH KFPVFMGRVY

DPRA

Sequence human C1-IA:

10 20 30 40 50

MASRLTLLTL LLLLLAGDRA SSNPNATSSS SQDPESLQDR GEGKVATTVI

60 70 80 90 100

SKMLFVEPIL EVSSLPTTNS TTNSATKITA NTTDEPTTQP TTEPTTQPTI

110 120 130 140 150

QPTQPTTQLP TDSPTQPTTG SFCPGPVTLC SDLESHSTEA VLGDALVDFS

160 170 180 190 200

LKLYHAFSAM KKVETNMAFS PFSIASLLTQ VLLGAGENTK TNLESILSYP

210 220 230 240 250

KDFTCVHQAL KGFTTKGVTS VSQIFHSPDL AIRDTFVNAS RTLYSSSPRV

260 270 280 290 300

LSNNSDANLE LINTWVAKNT NNKISRLLDS LPSDTRLVLL NAIYLSAKWK

310 320 330 340 350

TTFDPKKTRM EPFHFKNSVI KVPMMNSKKY PVAHFIDQTL KAKVGQLQLS

360 370 380 390 400

HNLSLVILVP QNLKHRLEDM EQALSPSVFK AIMEKLEMSK FQPTLLTLPR

410 420 430 440 450

IKVTTSQDML SIMEKLEFFD FSYDLNLCGL TEDPDLQVSA MQHQTVLELT

460 470 480 490 500

ETGVEAAAAS AISVARTLLV FEVQQPFLFV LWDQQHKFPV FMGRVYDPRA
